# The Collargol Treatment

**Published:** 1909-09

**Authors:** 


### THE COLLARGOL TREATMENT.

WRITING from Lazarus's Division of the Krankenhaus d. jüd. Gemeinde in Berlin, Fabian and Knopf (*Berl. klin. Wochenschr.*, July 26, 1909), describe twenty-five cases subjected to collargol treatment. Fifty c.c. of a 1 per cent. collargol solution (½ gram of the dry substance) were given by enema twice daily, generally for eight days. When enemata were not well retained, the remedy was given per os (150 c.c. of a 1 per cent. solution in cacao per day, in three doses).

Summarised, the results were: one case of post-scarlatinal rheumatism, eight enemata of each 25 c.c. 1 per cent. collargol; after the third, reduction of pain; after the seventh, temperature normal, no more pains; cured.

Three cases of gonorrheal arthritis. The first required eleven enemata of each 50 c.c. 1 per cent. collargol; temperature became normal after the fourth; pains finally disappeared; cure permanent; heart intact. The second got 16 enemata; swelling disappeared after the sixth, pain after the last. The third got sixteen enemata (case of gonitis, iritis and scleritis gonorrheica); remission of pains and temperature; after five further collargol enemata, temperature became quite normal; cure complete.

Fourteen cases of acute rheumatic polyarthritis. Ten cured with collargol, while the four others did better with aspirin. They conclude that collargol should be used in cases where the salicylates fail or are not well borne. In their opinion its action is less rapid than that of salicylates; but it must be remembered that they did not administer collargol by the most energetic method—intravenous injection—and that, moreover, the dose they gave rectally was rather small.

Two cases of colicystitis with urinary sepsis. The first got 1 per cent. collargol by mouth for nine days, 150 c.c. daily; also bladder irrigations with boric acid and silver nitrate. On the fifth day the temperature was normal; the bacteria almost entirely disappeared from the urine and the leucocytes also became less in number. Patient discharged in excellent condition. The second got twelve collargol enemata with salol internally. Gradual apyrexia; urinary findings approximately normal; abundant gain in weight; cured.

Five cases of streptococcic and staphylococcic septicemia. One was a scarlatinal sepsis with bronchopneumonia, adenophlegmon; collargol enemata; death. The second was an ulcerative endocarditis with septicemia, acute hemorrhagic nephritis, septic retinitis; collargol enemata also of no avail. The third was a post-abortion sepsis; collargol enemata not retained; death. The fourth was a septicemia with spleen tumor, cyanosis, chills, 105°F.; collargol enemata; death. The fifth was a septic endocarditis and staphylococcemia after post-diphtheritic angina. Collargol in cocoa by mouth (50 to 100 c.c. daily) for fourteen days, as enemata were rejected. Incision of fluctuating swelling in the region of right parotis; successive remission of temperature, cessation of joint pains, disappearance of pericardic noises, but persistence of loud whistling murmur on other ostias. After withdrawal of collargol, pulse was regular but rapid; occasionally precordial anxiety. Relapse about 15 days after stopping collargol. Twice daily 50 c.c. of 1 per cent. collargol injected rectally for eleven days, the enemata being now retained. Remission of fever and pain. Upon discharge, no cardiac murmur, no joint pain, pulse a little rapid, cardiac boundaries approximately normal, cure complete.

The effect of collargol is generally first to diminish the pain, while temperature only gradually returns to normal.

